# Patterns of presentation, prevalence and associated factors of mortality in ICU among adult patients during the pandemic of COVID 19: A retrospective cross-sectional study^☆^

**DOI:** 10.1016/j.amsu.2022.103618

**Published:** 2022-04-14

**Authors:** Shimelis Seid, Habtu Adane, Getachew Mekete

**Affiliations:** a Department of Anesthesia, College of Health Sciences, School of Medicine, Debre Tabor University, Debre Tabor, Ethiopia; b Department of Anesthesia, College of Medicine and Health Science, University of Gondar, Gondar, Ethiopia

**Keywords:** Admission, Adult patient outcome, Intensive care, Mechanical ventilation, Mortality, COVID-19

## Abstract

**Background:**

There is concern that patients admitted to the intensive care unit (ICU) with Corona Virus Disease 2019 (COVID-19) have variable prevalence reports of mortality. The survival rates are also inconsistently reported due to varying follow-up periods. Even if data on outcomes and baseline characteristics of ICU patients with COVID-19 is essential for action planning to manage complications, it is still left undisclosed in our study setting.

**Materials and method:**

This cross-sectional study was conducted on 402 samples using a retrospective chart review of patient's data who were admitted in the past 2 years of the adult ICUs. All the data were entered and analyzed with SPSS version 21. A multivariable Logistic regression analysis was used to identify the association between outcome variables with independent factors and a p-value of less than 0.05 was taken as statistically significant with a 95% confidence interval. We used text, tables, and figures for the result.

**Result:**

The overall prevalence of mortality among adult patients admitted to ICU during COVID-19 pandemics was 67.4%. From the multivariable logistic regression analysis, factors that were shown to have an association with an increase in ICU patient mortality were; lack of Vasopressor support, patients who had confirmed COVID 19 infection, core body temperature at admission greater than 37.5 °c, SPO2 at admission less than 90%, patients who had diagnosed ischemic heart disease (IHD), patients with acute respiratory distress syndrome (ARDS), patients who were intubated and mechanically ventilated (MV), and patient's ICU length of stay longer than two weeks.

**Conclusion:**

The prevalence of ICU mortality in adult patients was higher in Debre Tabor Comprehensive specialized hospital. Therefore, clinicians need to minimize factors that maximize patient mortalities like ARDS, hyperthermia, Desaturation, Covid infection, IHD, intubation and MV, lack of Vasopressor use, and prolonged ICU stay.

## Highlights

- The prevalence of mortality in ICU was 67.4%
- Hyperthermia increases mortality in ICU
- Use of vasopressor in ICU decreases mortality

## Acronym and abbreviations

ARDS =: Acute Respiratory Distress Syndrome
BMI =: Body Mass Index
CI: Confidence Interval
COPD =: Chronic Obstructive Pulmonary Diseases
COVID =: Corona Virus Disease
˚C: Degree Centigrade
GCS =: Glasgow Comma Scale
ICU =: Intensive care unit
IHD =: Ischemic Heart Disease
MAP: Mean Arterial Pressure
mmHg =: Millimeter Mercury
MV =: Mechanical Ventilation
SARS-COV2 =: Severe Acute Respiratory Syndrome Corona Virus 2
SPO2 =: Saturation of Peripheral Arterial Oxygenation

## 1 Introduction

The intensive care unit is the place that provides special support to the vital organ of patients and utilizes the skill of nursing, physicians, anesthetists, and other staff experienced in the management of the problems [1,2]. Severe pulmonary disease caused by severe acute respiratory syndrome coronavirus 2 (SARS-CoV-2), has devastated many countries around the world. The priorities of many institutions have changed to manage critically ill coronavirus infectious disease-2019 (COVID-19) patients and overwhelmed the medical, economic, and social system. Especially, in the intensive care unit, which is the primary area of patient admission, affects the work style of many professionals [3,4]. Regarding its mode of transmission, people usually become infected with the coronavirus when they breathe in microdroplets and air droplets from infected individuals into their respiratory tract [4].

It is estimated that 15–20% of patients who were positive for COVID 19 require hospitalization and 3–5% of them need critical care admission [5,6]. As the study showed, patients admitted to ICU with COVID-19 have variable prevalence reports of mortality, which range from 17% to 88% [7]. Again, the survival rates are inconsistently reported due to varying follow-up periods. Recent reports from the USA, Italy, and China indicate the prevalence of mortality among ICU patients was between 60%–85% with data on definite outcomes [[8], [9], [10]]. In another study from England, Wales, and Northern Ireland regarding the 28 days of ICU covid-19 patients, the prevalence of ICU mortality showed from 43.5% before the first pandemic period to 34.3% after the COVID 19 pandemic [11]. Another study from Turkey also showed that the mortality rate was very high in patients with comorbid diseases such as pulmonary infection with COVID 19, cardiac disease, diabetes mellitus, and solid organ transplant patients [12]. In Sub -Saharan Africa, the mortality rate of COVID-19 patients in ICU showed 60.4% in Libya [13], 24% in Egypt [14], and 46.8% in southern parts of Ethiopia [15].

Since the first cases were diagnosed in Ethiopia on March/13/2020, both the rate of ICU admission and the spread of SARS-COV-2 infections are increasing which results in unprecedented strain on ICU healthcare delivery [16,17]. The mortality prevalence variation across different continents was explained by differences in patient characteristics and socio-economic status, ICU admission thresholds, health care systems, and availability of variable numbers of ICU beds between hospitals [18]. Data on outcomes and baseline characteristics of ICU patients with COVID-19 are essential for action planning to manage complications and to assess the need for rehabilitation in ICU survivors [19].

The severity of COVID-19 signs and symptoms depends on the presence of different risk factors such as; advanced age, having comorbidities such as hypertension, pneumonia, COPD, ARDS, diabetes mellitus, tuberculosis, renal disease, hepatic disease, cardiac disease, and behavioral factors such as substance abuse and smoking [3,20,21]. Understanding the most determinant factors of ICU outcome is crucial both for shared decision making and surge planning.

Patients admitted to the intensive care unit had a longer length of stay and higher incidence of respiratory failure, ARDS requiring invasive mechanical ventilation, shock, ischemic heart diseases, acute kidney injury requiring dialysis, and mortality compared with patients in the general practice unit [22,23].

There must be adequate availability of basic requirements such as trained personnel, oxygen, medications, therapy and diagnostic equipment, safe transportation, and reliable power supply in ICU to care for critically ill patients [24]. However, there is a greater burden of little infrastructure availability in developing countries to care for critically ill patients [25]. This problem is also pronounced, especially in the era of COVID 19.

There are limited previous studies conducted in those study areas regarding the patterns of patient presentation, prevalence, and associated factors of mortality in adult ICUs. To help address the growing concern of critical illness, we have conducted a retrospective cross-sectional study of critically ill patients during the COVID-19 pandemic time. This helps us to identify the current gap and to improve quality services. It will also help to initiate to do other studies and develop local guidelines in the study area.

### 1.1 Objective

To identify the Patterns of presentation, prevalence, and associated factors of mortality in ICU among adult patients during the pandemic of COVID 19 at Debre Tabor Comprehensive Specialized Hospital from March 13, 2020 to January 15/2022.

## 2 Methods and materials

After we got ethical approval from Debre Tabor University ethical review committee, a cross-sectional study was conducted on 402 adult patients using a retrospective chart review from a record routinely completed by nursing staff. All pieces of information regarding ICU admissions and discharges during the COVID 19 era were documented. To ensure the clarity and validity of the assessment tool, three experts from senior health professionals reviewed and pre-tested it in the ICU outside our study area. Data collectors were two well-trained senior Nurses, who were working in the ICU. Demographic data and information regarding diagnosis, outcome, and length-of-stay were drawn within two years from 13 March 2020 to 15 January 2022. This study is already registered at research registry.com with a unique identifying number (UIN): researchregistry7645. This study is also fully compliant with the STROCSS 2021 criteria [26].

### 2.1 Study area

Debre Tabor comprehensive, specialized hospital is located in Amhara, regional state, Northern parts of Ethiopia and it is 666 km far from the central city, Addis Ababa. It was established in 1923 and is the only comprehensive, specialized hospital in south Gonder ᴢone. It offers health care services for more than 1.3 million people. This hospital gives curative, preventive, and rehabilitative care services to the community which has also three central ICUs, one of them is pediatrics and the other two adult ICUs. On average, the ICU centers have 8–12 beds with respective mechanical ventilators and integrated non-invasive monitors.

### 2.2 Inclusion and exclusion criteria

In our study setting, Patients age 15 years and above are usually admitted to adult ICUs. We have included all patients age 15 years and above who were admitted to ICUs so as not to miss important information. The exclusion criteria were; those with incomplete data on the charts and with missed patients’ charts. Other patients excluded from this retrospective study were; those who died immediately and within 2 hours of admission. This is due to a lack of sufficient time to deliver optimal care in the ICU, and the outcomes of these patients are related to other ward care.

### 2.3 Variables of the study

#### 2.3.1 Dependent variable

Patterns of presentation and prevalence of mortality.

#### 2.3.2 Independent variables

Sociodemographic factors (sex, age, BMI, occupation, income, marital status, educational status, residence), vital sign status during admission, diagnosis type at admission, COVID 19 status, presence of other comorbid illness, source of admission, frequency and category of admission, interventions, and Length of ICU stay.

### 2.4 Sample size, sampling techniques, and analysis

**Fig. 1:**
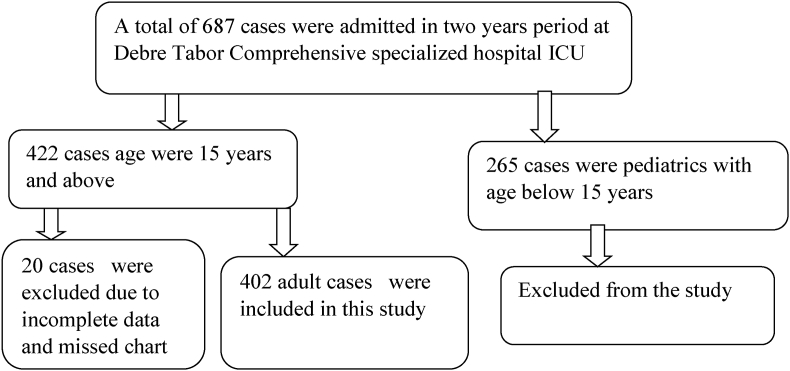
A flowchart diagram showing patient enrolment procedures from Debre Tabor Comprehensive Specialized Hospital ICU.

All consecutively admitted patients from March 2020 to January 2022 at Debre Tabor Comprehensive specialized hospital adult ICUs were included. Patient enrollment procedures were shown below (Fig. 1).

#### 2.4.1 Data analysis and interpretation

All the data were entered and analyzed with SPSS version 21. The data were presented as a mean ± standard deviation for the data that were normally distributed or as a median ± interquartile range for data that were non-normal distribution. Hosmer-Lemeshow goodness-of-fitness test was used to check model fitness. Crude odds ratios and adjusted odds ratios with their 95% confidence intervals were estimated in the bivariable and multivariable logistic regression analysis to identify the association between each independent variable and outcome variables, respectively. After bivariable binary logistic regression analysis, variables that had P-values of less than 0.2 were entered into multivariable logistic regression. From multivariable logistic regression, a p-value of less than 0.05 was taken as statistically significant with a 95% confidence interval. Descriptive statistics, figures, and tables were used for the result.

## 3 Result

### 3.1 Sociodemographic characteristics of study participants

From the two ICUs included, a total of 422 patient charts were selected. Among the collected data, 4.74% of study participants’ charts were excluded from analysis due to incomplete information. The median ± interquartile ranges of both ages and BMI of study participants were 60 ± 34.6 years and 24.3 ± 6.6 kg/m^2^ respectively. Among the total patients included in this study, 233(58%) were males and 169(42%) were females. Regarding their educational status, 158(39%) can read and write, and 82 (20.4%) were illiterates. Regarding their occupational status, 30.3% were government employed, 29.4% self-employed and 18.7% were unemployed. Among the study participants, 231(57.5%) were from rural areas and 171(42.5%) came from urban areas (see Table 1).

**Table 1:** Sociodemographic and clinical characteristics of patients admitted to adult ICUs at Debre Tabor Comprehensive specialized hospital, from March 13/2020 to January 15/2022.

Variables	Category	Frequency (n)	Percentage (%)
age	15–50 years	167	41.5%
	>50 years	235	58.5%
BMI	<18.5 kg/m^2^	73	18.2%
	18.5–24.9 kg/m^2^	171	42.5%
	≥25 kg/m^2^	158	39.3%
Sources of admission	From operation theatre	82	20.4%
	From Emergency Department	198	49.3%
	From other wards	122	30.3%
GCS status at admission	Conscious	260	64.7%
	Unconscious	142	35.3%
SPO2	>95%	32	8%
	90–95%	88	21.9%
	<90%	282	70.1%
MAP	65–106 mmHg	99	24.6%
	<65 mmHg	149	37.1%
	>106 mmHg	154	38.3%
Pulse Rate	60-100 beats per minute	79	19.7%
	<60 beats per minute	103	25.6%
	>100 beats per minute	220	54.7%
Respiratory rate	12-20 breaths per minute	68	16.3%
	<12 breaths per minute	12	3%
	>20 breaths per minute	322	80.1%
Temperature status	36.5–37.5 °C	64	15.9%
	<36.5 °C	205	51%
	>37.5 °C	133	33.1%

BMI=Body Mass Index; GCS = Glasgow Coma Scale; MAP = Mean Arterial Pressure.

### 3.2 Clinical comorbidities diagnosed during ICU admission

The most common indications for ICU admission were respiratory diseases 39.5%, followed by cardiovascular diseases 34%, and endocrine diseases (12.3%). Among the respiratory diseases, COVID 19 induced ARDS was the commonest cause of admission (46%), followed by Pneumonia (21%), acute exacerbated asthma (15%), and post-traumatic airway obstruction (10%). Other comorbidities like COPD, pneumothorax, and airway burn cover 8%. Regarding the cardiovascular causes of ICU admission, IHD was the commonest factor (52%), followed by CHF (26%) and Septic shock (19%). Other factors like valvular heart diseases, cardiac arrest, and pericardial effusion shared the remaining percentages (3%). Among the endocrine diseases, diabetic Ketoacidosis was the major endocrine cause for admission in 66% of cases. Stroke and Head injury were the dominant factors of admission from the neurologic diseases group in 35% and 23% of cases respectively (see Table 2).

**Table 2:** Shows the distribution of admission causes among adult patients admitted to adult ICUs in Debre Tabor Comprehensive Specialized Hospital, Northern-Ethiopia.

Comorbidities	Frequency (n)	Percentages (%)
Respiratory diseases	159	39.5%
Cardiovascular diseases	137	34%
Endocrine diseases	49	12.3%
Neurologic diseases	25	6.2%
Renal diseases	13	3.3%
Hematologic diseases	10	2.6%
Others	8	2.1%
Total	402	100%

### 3.3 Patterns of oxygenation, medication, and their outcome in ICU

**Fig. 2:**
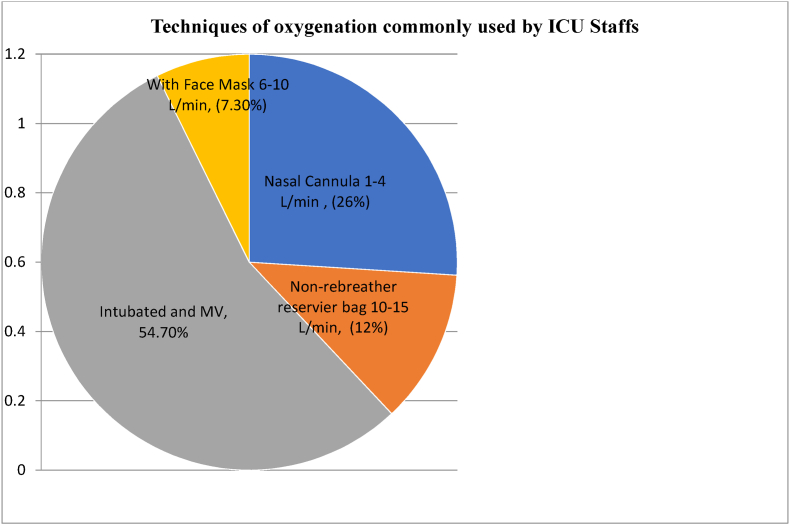
Shows the patterns of oxygenation after admission to adult ICU patients at Debre Tabor comprehensive specialized hospital, Northern-Ethiopia.

During admission to ICU, most of the patients need oxygenation using different mechanisms. Among the total patients who need oxygen, 26% of cases were supported by non-re-breathing masks and 36.8% of admitted cases were supported with vasopressors (specifically, with adrenaline and dopamine). Among the 402 ICU patients with data on definite outcomes, ICU length of stay was 14 (Interquartile range: 8–20) days. Among them; 220 (54.72%) received mechanical ventilation, 271(67.4%) died and 131 (32.6%) were discharged. Steroid medications like dexamethasone and hydrocortisone were given immediately after admission for indicated cases. The commonest antibiotics given were Ceftriaxone, Azithromycin, and Meropenem (see Fig. 2).

### 3.4 Prevalence of mortality and associated factors in ICU

The overall mortality of patients admitted to adult ICUs during COVID-19 pandemics was 67.4% (95% CI: 62.9–72.1). From the multivariable logistic regression analysis, factors that were shown to have an association with an increase in mortality were; patients who weren't on vasopressor support (AOR: 7.82; CI = 2.20–27.82), patients who had confirmed Covid 19 infection (AOR: 27.1; CI = 5.69–128.7), the Core temperature at admission greater than 37.5 °c (AOR: 7.68; CI = 2.01–29.27), SPO2 at admission less than 90% (AOR: 0.17; CI = 0.03–0.85), patients who had diagnosed IHD (AOR: 15.79; CI = 5.11–48.78), ARDS patients (AOR: 34.7; CI = 10.69–113.1), patients who were intubated and mechanically ventilated (AOR: 7.17; CI = 2.43–21.14), and patient's ICU length of stay longer than two weeks (AOR: 3.37; CI = 1.15–9.88) (see Table 3).

**Table 3:** Shows factors associated with outcome variables during bivariable and multivariable logistic regression analysis at Debre Tabor comprehensive specialized hospital adult ICUs (n = 402).

Variables	Category	Patient outcome		COR (95% CI)	AOR (95% CI)	*P*-value
		Death n(%)	Alive n(%)			
Age	15–50 years	124(45.8%)	43(32.8%)	1	1	
	>50 years	147(54.2%)	88(67.2%)	1.73(1.12, 2.67)	1.34(0.51, 3.52)	0.559
Vasopressor given	No	143(52.8%)	111(84.7%)	4.97(2.92, 8.46)	7.82(2.20, 27.82)	**0.001**
	Yes	128(47.2%)	20(15.3%)	1	1	
Frequency of admission	First admission	260(95.9%)	108(82.4%)	1	1	
	Readmission	11(4.1%)	23(17.6%)	5.03(2.37, 10.69)	1.46(0.30, 7.05)	0.634
Patient status for COVID 19 at admission	Suspected for COVID 19	12(4.4%)	92(70.2%)	1	1	
	Confirmed	259(95.6%)	39(29.8%)	50.92(25.55, 101.44)	27.1(5.69, 128.7)	<**0.001**
MAP	65-106 mmhg	75(27.7%)	24(18.3%)	1	1	
	<65 mmhg	77(28.4%)	72(55%)	1.08(0.60, 1.97)	3.25(0.86, 12.23)	0.082
	>106 mmhg	119(43.9%)	35(26.7%)	3.18(1.94, 5.22)	2.96(7.35, 11.91)	0.127
Temperature at admission	36.5–37.5 °c	32(11.8%)	32(24.4%)	1	1	
	<36.5 °c	125(46.1%)	80(61.1%)	6.0(3.01, 11.96)	3.79(0.45, 32.04)	0.222
	>37.5 °c	114(42.1%)	19(14.5%)	3.84(2.19, 6.73)	7.68(2.01, 29.27)	**0.003**
SPO2 at admission	>95%	28(10.3%)	4(3.1%)	1	1	
	90–95%	73(26.9%)	15(11.5%)	1.44(0.44, 4.71)	0.53(0.09, 3.09)	0.477
	<90%	170(62.7%)	112(85.5%)	4.61(1.58, 13.50)	0.17(0.03, 0.85)	**0.032**
IHD at admission	normal	195(72%)	32(24.4%)	1	1	
	Diagnosed	66(24.4%)	91(69.5%)	8.40(5.15, 13.72)	15.79(5.11, 48.78)	**<0.001**
	Undiagnosed	10(3.7%)	8(6.1%)	4.88(1.79, 13.28)	0.91(0.04, 20.33)	0.951
ARDS at admission	Yes	106(39.1%)	115(87.8%)	11.2(6.28, 19.92)	34.7(10.69, 113.1)	**<0.001**
	No	165(60.9%)	16(12.2%)	1	1	
Intubated and MV	Yes	102(37.6%)	118(90.1%)	15.04(8.06, 28.1)	7.17(2.43, 21.14)	**<0.001**
	No	169(62.4%)	13(9.9%)	1	1	
Length of stay	>2 weeks	169(62.4%)	100(76.3%)	1.95(1.21, 3.12)	3.37(1.15, 9.88)	**0.027**
	<2 weeks	102(37.6%)	31(23.7%)	1	1	

MAP = Mean Arterial pressure; SPO2=Saturation of Peripheral arterial Oxygenation; IHD = ischemic heart disease; ARDS = acute respiratory distress syndrome; MV = Mechanical Ventilation; COVID=Corona Virus Disease; COR=Crude Odd Ratio; AOR = Adjusted Odd Ratio.

## 4 Discussion

Our study aimed to identify the patterns of presentation, prevalence, and associated factors of mortality in ICU among adult patients during the pandemic of COVID 19 at Debre Tabor Comprehensive Specialized Hospital. As the COVID-19 pandemic continues, knowing the clinical outcomes of ICU admitted patients are critically important for clinicians to prioritize resources and triage patients in a highly congested environment.

In our study, the overall mortality of patients admitted to ICUs in the era of COVID 19 was 67.4%. Factors associated with increased mortality in ICUs were; confirmed COVID 19 patients at admission, patients who weren't on vasopressors, body core temperature >37 °c, peripheral arterial oxygen saturation <90% at admission, patients with diagnosed IHD, ARDS patients, intubated and mechanically ventilated patients, and patient's length of stay in ICU > two weeks.

As our study result showed, respiratory diseases were the commonest causes of ICU admission (39.5%), followed by cardiovascular diseases (34%), endocrine diseases (12.3%), neurologic diseases (6.2%), and renal diseases (3.3%). The admission patterns in this study result is supported by a study done in China, which showed that the majority of deaths were due to respiratory failure (88.5%) followed by myocardial damage (62.8%). Based on the above study, the commonest complications among the ICU patients included were acute respiratory distress syndrome (ARDS) (97.4%) and renal damage (38.5%) [27]. Based on the degree of hypoxemia, ARDS is classified as mild, moderate, and severe. Patients with moderate to severe levels of ARDS usually require intubation and invasive mechanical ventilation. A global literature survey study showed that the mortality prevalence among mechanically Ventilated COVID 19 patients in ICU was 59% and in ARDS patients, 45% [28]. There are also reports regarding a strong association between worse clinical outcomes and hypoxemia. A study with COVID-19-associated pneumonia found that peripheral arterial oxygen saturation >90% predicted survival in ICU with a specificity of 97.2% and sensitivity of 84.6% [29].

The prevalence of mortality in our study was comparable with studies done in Washington state, which was 67%, and in Nigeria 68.4% [30].

Mortality prevalence in this study was higher compared with a study done in Georgia 35.7% [31], Sweden 17.8% [32], Switzerland 7% [33], Netherland 29% [34], China 4.3% - 61.5 [35,36], Poland 42% [37], Libya 60.4% [38], in Egypt 24% [39], and in southern parts of Ethiopia 46.8% [40]. The possible justifications for this variation in mortality prevalence might be limited access to resources, variation in the study setting and quality of service delivery.

Mortality prevalence in this study report was lower compared with studies done in Brazil, 86.7% [41], and New York city 88.1% [42]. The reason could be the late incidence of the diseases, which gives an advantage for early preparation before the outbreak and implementation of guidelines [37,38].

As our study result showed, ICU patients who were intubated and mechanically ventilated were 7.17 times more likely to have higher mortality risks compared with non-intubated patients. This result was supported by another study stating that mortality among intubated and MV patients was higher compared with non-intubated cases [43]. This result is also supported by a cross-sectional study from Ethiopia [15]. The possible justifications could be intubation and mechanical ventilation maximizes ventilator-associated infection, immobilization, and the potential for long-term physical and neuro-cognitive dysfunction [5].

The result of our study also showed that patients who had peripheral arterial oxygen saturation below 90% at ICU admission were more likely to die compared with patients who had saturation levels above 90%. In line with this result, a study done in Peru and Mexico showed that ICU patients who had saturation below 90% were 7.74 and 4.48 times more likely to die in ICU compared with those who had saturation above 90%, respectively [40,41]. The justification could be due to clinical events like acute hypoxemia, which enhances cytotoxic functions of neutrophils and can induce inflammation. This will result in increased vascular permeability and elevated serum cytokine levels, which contribute to progressive lung damage and death [44].

Similarly, this study result showed that ICU admitted patients who had tested positive for COVID 19 virus earlier were 27.1 times more likely to die in the ICU compared with patients who were not confirmed for SARS COV2 infection. This result was supported by other studies made in the USA [45]. The justifications could be due to having different predictors on the outcomes of critical patients admitted to ICU. Superimposed infection with COVID-19 results in increased mortality risks due to virus-activated “cytokine storm syndrome” and COVID 19 virus effects on the heart, which is called ‘’fulminant myocarditis'’, compared with non-COVID critical patients [46].

On the other hand, our study's result showed patients who were diagnosed with ischemic heart diseases at admission were 15.79 times more likely to die in the ICU compared to those patients without IHD. Consistent with this result, cohort studies done in Pakistan and Egypt showed patients with IHD at admission were 13.04 times riskier to develop a death in the ICU compared with patients without signs of IHD [14,47]. Compared with patients without cardiac injury, patients with COVID 19 induced cardiac injury presented with a more severe acute illness manifested by abnormal higher levels of C-reactive protein, increased troponin levels, increased serum creatinine, and radiographic findings like multiple mottling and ground-glass opacity, which maximizes ICU patients mortality rates [48].

Our study's results also showed that ICU admitted patients with the diagnosis of ARDS were highly likely to develop mortality in the ICU compared to patients admitted with non-ARDS diseases. In line with this study, a study done in Germany showed an increase in mortality among the ARDS patient group [[49], [50], [51]]. This result is also supported by studies done in California [52], Spain [53], and France [54]. The possible reasons for an increase in the prevalence of mortality among COVID-19-related ARDS patients were due to having reduced respiratory system compliance together with increased D-dimer concentrations compared with non-COVID ARDS patients [55].

Similarly, the present study result showed that ICU admitted patients who had a length of stay of more than two weeks were 3.37 times more likely to die in ICU compared with other patients who stayed less than 2 weeks in the ICU. The present study was supported by a study done in Canada that showed a positive association between prolonged ICU stay with increased mortality rate [56,57]. The possible reasons could be prolonged stay in ICU increases the risk of hospital-acquired infection, pulmonary embolism, DVT, sepsis, pneumonia, and malnutrition; all further promote devastating patient outcomes [58].

As this study result showed, ICU admitted patients who had a core body temperature, record above 37.5 °c were 7.68 times more likely to die in the intensive care compared with patients who had a record of below 37.5°c. The results of this study are supported by cohort studies done in Chicago, and Singapore showed that ICU patients with a body temperature above 103.3 °F were more likely to die in the ICU compared with normothermic and hypothermic patients [59]. Based on the above study, the prevalence of hyper-thermic patients’ mortality in ICU was 61.1% [58]. Hyperthermia in critically ill patients increases hemodynamic instability inhibits enzymatic function, and increases the risk of infection and immune comptonization. All factors result in further increased morbidity and mortality risks for the patients [7,59].

### 4.1 Limitations of this study

Being a retrospective and single-center study, having limited sample sizes, and heterogeneous study populations were the limitations of this study.

### 4.2 Conclusion and recommendation

The commonest causes of adult ICU admission were respiratory and cardiovascular diseases. The prevalence of mortality in adult patients was higher in Debre Tabor Comprehensive specialized hospital ICUs. Therefore, clinicians need to minimize factors that increase patient mortalities like; ARDS, hyperthermia, desaturation, Covid infection, IHD, intubation and MV, lack of vasopressor use, and prolonged ICU stay. We recommend for researchers to state with a longer follow up period and at the national level in the future.

## Funding source

None.

## Author contributions

**Mr. Shimelis Seid** has contributed to the preparation of a proposal, development of a questionnaire, study designing, conceptualization, supervising data collection, data entry, data analysis, data interpretation, and final edition of this manuscript. **Mr. Habtu Adane** has helped with the supervision of data collection, data analysis, and final output writing and participated in the preparation of this manuscript for submission in this journal. **Mr. Getachew Mekete** has contributed with designing, conceptualization, supervision of data collection and edition of the manuscript.

## Data availability

All data generated or analyzed during this study are included in this article and found on a reasonable base.

## Registration of research studies

Name of the registry: research registry.

The unique identifying number of registration ID: researchregistry7645.

Hyperlink to your specific registration (must be publicly accessible and will be checked): https://www.researchregistry.com/browse-the-registry#home/

## Guarantor

Email: shemsu864@gmail.com.

+251975184808.

## Provenance and peer review

This study is externally peer-reviewed and not commissioned.

## Declaration of competing interest

None.
